# Treatment of Refractory Postural Tachycardia Syndrome with Subcutaneous Octreotide Delivered Using an Insulin Pump

**DOI:** 10.1155/2015/545029

**Published:** 2015-05-19

**Authors:** Muhammad Khan, Jing Ouyang, Karen Perkins, John Somauroo, Franklin Joseph

**Affiliations:** ^1^Department of Diabetes & Endocrinology, Countess of Chester Hospital NHS Foundation Trust, Chester CH2 1UL, UK; ^2^Department of Cardiology, Countess of Chester Hospital NHS Foundation Trust, Chester CH2 1UL, UK

## Abstract

Postural Tachycardia Syndrome (PoTS) represents a disorder of the autonomic nervous system that results in symptoms of orthostatic intolerance. Despite having a severe impact on the patient's quality of life, the current treatment options for PoTS are based on limited evidence. Subsequently, this results in clinicians having to utilise a variety of treatment regimens in the hope of successfully providing symptomatic relief. However, the options available for PoTS are not without significant side effects that can worsen an already debilitating condition. Our cases provide a further novel treatment option for clinicians to consider in PoTS refractory to established treatments.

## 1. Introduction

Postural Tachycardia Syndrome (PoTS) represents a subset of dysautonomic conditions characterised by features of orthostatic intolerance and tachycardia, but without the presence of orthostatic hypotension [1]. Formal guidelines on the management of PoTS are lacking, yet the sparse literature on this condition suggests several possible treatment options. One such pharmacological agent is the somatostatin analogue octreotide. Although not specifically licensed for the treatment of PoTS, the limited literature outlining its efficacy makes it a useful adjunct for the symptomatic control of PoTS. However, the use of octreotide can be expensive, can be associated with debilitating side effects, and can cause inconvenience due to the required frequency of injections. We present two cases of PoTS inadequately controlled with existing treatments that included octreotide. Both patients eventually benefited from the subcutaneous octreotide delivered using an insulin pump. We believe this to be the first reported instance of this means of administration.

## 2. Case Report

### 2.1. Case Presentation

#### 2.1.1. Case  1

A 24-year-old woman presented with sudden onset of recurrent light-headedness and severe orthostatic intolerance relieved with recumbence following a pyrexial illness. She complained of light-headedness, nausea, heat intolerance, breathlessness, and fatigue but no palpitations. Initial physical examination reported no abnormalities except for a regular resting heart rate of 99 beats per minute (bpm) and soft systolic murmur. Biochemical investigations, chest X-ray, and an electrocardiogram revealed no abnormalities. Holter monitoring identified a sinus rhythm alternating with sinus tachycardia, thus raising the possibility of an underlying diagnosis of PoTS. Diagnostic tilt table testing (TTT) was ordered and confirmed the diagnosis of PoTS due to an increase in the patient's heart rate from 88 bpm at supine position to 122 bpm within 10 minutes of standing in the absence of orthostatic hypotension (supine blood pressure: 136/90 mmHg; blood pressure within 10 minutes of standing: 148/103 mmHg).

Initial management included an increase in both fluid intake to 3 litres per day and salt intake through slow sodium MR (600 mg, 10 per day). Medications including fludrocortisone (300 *μ*g once daily), bisoprolol (2.5 mg twice daily), paroxetine (20 mg once daily), midodrine (5 mg three times daily), and ivabradine (7.5 mg twice daily) were trialled, both serially and then in combination, but each proved to be ineffective. Subcutaneous (SC) octreotide was trialled with the dose increasing from 25 *μ*g twice daily to 50 *μ*g at 90-minute intervals, six times a day (300 *μ*g/24 h). Despite symptomatic relief, she reported excruciating abdominal cramps and episodes of diarrhoea occurring invariably following each injection. The escalating orthostatic symptoms resulted in her being wheelchair dependent and the progressive decline in her quality of life culminating in her being forced to withdraw from her job and suffering an episode of clinical depression. The severity and frequency of side effects prompted the notion of possibly administering SC octreotide via an insulin pump.

#### 2.1.2. Case  2

A 14-year-old girl presented to her general practitioner with a year long history of intermittent headaches associated with palpitations, light headedness, nausea, dizziness, and “going pale” upon standing from a supine position. Additionally, she reported experiencing “tunnel vision” which preceded brief episodes of loss of consciousness. Initial examination noted pallor, regular resting heart rate of 80 bpm, blood pressure of 120/64 mmHg on sitting and 122/68 mmHg on standing, and a relative tachycardia that resolved on standing. All other examinations were normal. Laboratory investigations, echocardiogram, and MRI to rule out intracranial pathology were normal. 24-hour ambulatory electrocardiogram showed recurring intermittent sinus tachycardia that coincided with the patient experiencing symptoms. Due to the progressive worsening in the severity of her symptoms, the patient forcibly removed herself from extracurricular activities in school leaving her expressing low self-esteem and worthlessness due to the feelings of “vulnerability” upon standing. With her symptoms having such a negative impact on her life physically, socially, and psychologically, she became reclusive and isolated and was diagnosed with depression by the adolescent psychiatrists. Following review by a cardiologist the following year, the possibility of PoTS was considered. Diagnostic TTT was conducted which identified an elevation in her heart rate from 80 whilst supine to 140 bpm within 10 minutes of standing in the absence of orthostatic hypotension (supine blood pressure: 114/73 mmHg; blood pressure within 10 minutes of standing: 124/82 mmHg), thus confirming a diagnosis of PoTS.

Therapies such as an increase in fluids to 3 litres per day, slow sodium MR (up to 600 mg eight per day), fludrocortisone (up to 200 *μ*g once daily), and midodrine (maximum 5 mg three times daily; escalation of midodrine to 10 mg three times daily resulted in peripheral vasospasm and intermittent loss of sensation in both hands and feet) were sequentially trialled either as monotherapy or in combination with no resolution of symptoms. As the frequency of symptoms increased further, the patient felt “inhibited” by her symptoms, started to take excessive periods off school, and felt “immense dread” at the prospect of returning to school. With no effective symptomatic control, the patient was trialled on 25 *μ*g of SC octreotide, three times daily that was titrated to 50 *μ*g, six times daily (300 *μ*g/24 h); coverage for this method was obtained via an “individual patient funding request” to the local medicines management group. Although providing a degree of symptomatic control which allowed her to manage effectively at university, her quality of life was significantly hindered by the nausea and diarrhoea associated with each injection. Furthermore, she also reported bladder dysfunction, acne, hair loss, dysmenorrhoea, and intermittent vaginal bleeding following the initiation of octreotide. As a result of the adverse side effects from the intermittent injections, she underwent a trial of octreotide administered via an insulin pump.

### 2.2. Treatment

#### 2.2.1. Case  1

Following an initial period of education on how to use the insulin pump by a specialist diabetic/endocrine nurse, the patient received 10 *μ*g of octreotide per hour for a total of 12 hours (120 *μ*g/24 h) over a course of two weeks through the Animas 2020 insulin pump; coverage for this method was obtained in an identical manner as for patient 1. She replaced her reservoir (2.0 mL cartridge) on alternate days and the cannula and infusion set (inset II infusion set, 6 mm cannula; 60 cm (23′′) tubing) were replaced every third day.

#### 2.2.2. Case  2

After education on use of an insulin pump by a specialist diabetic/endocrine nurse, the patient received 11 *μ*g of octreotide per hour for a total of 12 hours (132 *μ*g/24 h) over a three-month period via the aforementioned insulin pump. Due to personal preference, the patient replaced both her infusion (inset II infusion set, 6 mm cannula; 60 cm (23′′) tubing) and cannula set on a daily basis.

### 2.3. Outcome

#### 2.3.1. Case  1

Following the two-week period, the patient reported a remarkable transformation in both physical and social functioning. She was no longer wheelchair dependent and was able to maintain an upright posture with no side effects. Additionally, she reported a significant improvement in her quality of life and given the vast improvement in her symptoms, she could return to work and conduct basic tasks without suffering from disabling orthostatic symptoms. To date, the patient reports no adverse effects, resulting from either her underlying PoTS or use of the insulin pump, and remains well controlled on a treatment regimen of SC octreotide, 120 *μ*g/24 h via an insulin pump.

#### 2.3.2. Case  2

At her 3-month review, the patient was completely transformed and elated. She reported a significant improvement in her functionality with none of the side effects she had experienced with the intermittent injections. The dysmenorrhea and acne resolved with the use of a third-generation oral contraceptive pill and her hair loss had improved; her intermittent vaginal bleeding being was attributed to the use of intermittent octreotide. Presently, the patient remains asymptomatic on a combination of 11 *μ*g per hour, for an average of 14 waking hours a day, of octreotide via an insulin pump in combination with 5 mg of midodrine three times daily, 200 *μ*g of fludrocortisone once daily, and 600 mg of slow sodium six per day. The patient reports no difficulties in using the insulin pump.

## 3. Discussion

Postural Tachycardia Syndrome (PoTS) is a heterogeneous group of disorders clinically defined in adults as a sustained rise in heart rate of ≥30 beats per minute (bpm) or an increase in heart rate to ≥120 bpm, within 10 minutes of movement from a supine to upright position or head-up tilt [1, 2]. However, differing diagnostic criteria exist for paediatric patients aged 14 or above whereby PoTS is defined as a rise in heart rate ≥40 bpm within 5 minutes of standing from supine position or head-up tilt [3]. Despite a number of cases and case series in the literature, there is presently no concrete epidemiological data regarding the exact prevalence of PoTS. However, PoTS is noted to primarily affect women (ratio of 5 : 1) who are predominantly young but the age range can extend between 15 and 50 years [4–6].

With such sparse information regarding the intricacies of this condition, PoTS reflects a syndrome that is commonly unfamiliar to the vast majority of physicians, thereby resulting in the condition being significantly underdiagnosed within the medical community. It is this lack of awareness of PoTS that unfortunately exposes patients with this condition to the various orthostatic symptoms described in our cases. Moreover, patients are left exposed to suffering from the loss in both physical and psychological functioning arising from PoTS due to the lengthy period of time between presentation and diagnosis.

Although the diagnosis of PoTS can be confirmed relatively easily with the use of tilt table testing (TTT), management of this condition, in the absence of established protocol or guidelines, can be challenging. However, there is evidence for a handful of both pharmacological and nonpharmacological adjuncts that can achieve symptomatic control of PoTS [1, 6]. These agents are not without adverse effects which in some scenarios, such as those described in our cases, can further impede the existing impaired quality of life [6]. In cases of PoTS refractory to established agents, octreotide is often utilised as the final therapeutic choice after other alternatives have been exhausted [7].

Octreotide is a somatostatin analogue which binds with high affinity to somatostatin receptor subtypes 2 and 5 [8]. The principal mechanism of action for this agent in the treatment of PoTS is to stimulate vasoconstriction in splanchnic vasculature, thus increasing venous return [6]. Presently, there are currently two methods of delivery by which octreotide can be administered to the patient. Short-acting (i.e., immediate release) octreotide is injected subcutaneously in the hip, thigh, or abdomen between 2 and 6 times per day. In contrast, long-acting octreotide analogue injections are delivered intramuscularly into the gluteal muscle once every 4 weeks [9].

Although the evidence base for use of octreotide in PoTS is substantially limited, the few trials conducted have identified it as a useful treatment option [7, 10, 11]. However, octreotide has side effects dependent on the method of administration. Intermittent subcutaneous injections result in nausea, abdominal pain, and diarrhoea; all of which occurred in the cases described. Furthermore, long-term administration has been associated with development of impaired glucose tolerance, biliary stasis, and, rarely, overt hyperglycaemia [8, 9]. In addition to this, use of present formulations of octreotide exposes patients to the physical discomfort and possible psychological stigma associated with repeated injection use. Another limitation to the use of octreotide is its financial implication. The annual costs can range from £1350 to £13,100, depending on the formulation and dosage used [9].

Our cases highlight a novel method of delivery to overcome the side effects of the intermittent subcutaneous injections as well as the cost of the analogue preparations. For our case series, we had internally calculated the 5-yearly cost of delivering octreotide via an insulin pump to be approximately £5200 (estimates used: £4000 every 5 years for the insulin pump, £1400 per year for consumables, and £3000 per year for octreotide (100 *μ*g/mL ampoules; 4 ampoules every 3 days); total cost over 5 years of £26000, total cost per year of £5200) which is substantially less than the estimated £24000 per year for long-acting release of octreotide at 40 mg every 3 weeks. Furthermore, as the total daily dosage required was lower compared to the dosage received via intermittent SC octreotide, and octreotide was only being delivered during waking ambulant hours, the patients' risk of long-term exposure is at least theoretically reduced. Finally, with fewer injections being required with this method, it reduces the burden of physical discomfort and the stigma associated with injecting.

Although insulin pumps are primarily used for the management of diabetes, their use has also been documented in other conditions such as Addison's disease [12]. With regard to their use in PoTS, there is anecdotal evidence to support this despite no literature presently documenting such claims. In our documented cases, both patients withdrew octreotide from a vial that was then inserted into the insulin cartridges for the corresponding insulin pump. Additionally, each patient received an hourly timed dose via the insulin pump during waking hours, approximately 12 hours for each patient, which was increased to 15 *μ*g during periods of physiological stress, for example, whilst exercising.

In cases refractory to established treatments, both physicians and patients can be overwhelmed by the prospect of no effective management option being available. Here, we report what we believe to be the first series of two such refractory cases of PoTS, where symptom relief was obtained by the novel delivery of octreotide via an Animas insulin pump. This technique provides physicians with another therapeutic option in the armamentarium against PoTS that clinicians may consider utilising in the management of this condition.
